# Time trends, frequency, characteristics and prognosis of short‐duration transient global amnesia

**DOI:** 10.1111/ene.14163

**Published:** 2020-02-26

**Authors:** M. Romoli, M. A. Tuna, L. Li, M. Paciaroni, D. Giannandrea, F. Tordo Caprioli, A. Lotti, P. Eusebi, M. G. Mosconi, M. Pellizzaro Venti, N. Salvadori, A. Gili, S. Ricci, F. Stracci, P. Sarchielli, L. Parnetti, P. M. Rothwell, P. Calabresi

**Affiliations:** ^1^ Centre for Prevention of Stroke and Dementia Nuffield Department of Clinical Neurosciences University of Oxford Oxford UK; ^2^ Neurology Clinic University of Perugia – S. Maria della Misericordia Hospital Perugia; ^3^ Neurology Unit Rimini ‘Infermi’ Hospital – AUSL Romagna Rimini; ^4^ Stroke Unit University of Perugia – S. Maria della Misericordia Hospital of Perugia Perugia; ^5^ Neurology and Stroke Unit USL Umbria 1 Gubbio and Città di Castello Hospital Perugia Italy; ^6^ Stroke Unit Addenbrooke's Hospital – Cambridge University Hospital Cambridge UK; ^7^ Public Health Department University of Perugia Perugia; ^8^ IRCCS ‘Santa Lucia’ Rome Italy

**Keywords:** transient global amnesia, stroke, epilepsy, seizure

## Abstract

**Background and purpose:**

Transient global amnesia (TGA) is characterized by a sudden onset of anterograde amnesia lasting up to 24 h. One major differential for TGA is transient epileptic amnesia, which typically lasts < 1 h. However, TGA can also be short in duration and little is known about the time trends, characteristics and prognosis of TGA cases lasting < 1 h.

**Methods:**

We compared the clinical features of TGA ascertained in two independent cohort studies in Oxfordshire, UK [Oxford cohort 1977–1987 versus Oxford Vascular Study (OXVASC) 2002–2018] to determine the time trends of clinical features of TGA. Results were validated in another independent contemporary TGA cohort in Italy [Northern Umbria TGA registry (NU) 2002–2018]. We compared the risk factors, clinical features and long‐term prognosis (major cardiovascular events, recurrent TGA and seizure/epilepsy) of patients presenting with episodes lasting < 1 h versus those lasting ≥ 1 h.

**Results:**

Overall, 639 patients with TGA were included (114 Oxford cohort, 100 OXVASC, 425 NU). Compared with the original Oxford cohort, there were more cases with TGA lasting < 1 h in OXVASC [32 (32.0%) vs. 9 (8.8%)] and NU (11.8% vs. 8.8% in Oxford cohort). In both OXVASC and NU, patient age, vascular risk factors and clinical features were largely similar between those with TGA lasting < 1 h versus those lasting ≥ 1 h. Moreover, there was no difference in the long‐term risk of seizure/epilepsy or major cardiovascular events between TGA lasting < 1 h versus TGA lasting ≥ 1 h.

**Conclusions:**

Short‐duration TGA episodes (<1 h) were not uncommon and were more frequent than in earlier studies. The clinical features and long‐term prognosis of short‐duration TGA did not differ from more typical episodes lasting ≥ 1 h.

## Introduction

Transient global amnesia (TGA) is characterized by sudden onset of anterograde amnesia lasting up to 24 h 1, 2. TGA was first reported in 1964 by Fisher and Adams 3 and the current clinical diagnostic criteria for TGA were proposed by Hodges and Warlow in 1990 2. However, little is known about the evolution of clinical features of TGA since then.

One diagnostic challenge of TGA is the exclusion of other potential causes of a transient amnestic syndrome, such as transient epileptic amnesia (TEA) 4. TEA is reported as being shorter than TGA, lasting typically <1 h 4, 5, 6, 7, 8 with higher rates of recurrence over time 4. However, up to 30% of TGA cases also recur 1 and some TGA cases can also present with electroencephalography (EEG) changes 9. More importantly, TGA cases can potentially be of short duration with 9% of the TGA cases reported to be < 1 h in the original 1990 cohort report 1, 2. Diagnostic certainty about TGA versus TEA will therefore partially depend on the frequency and prognosis of apparent short‐duration TGA cases among patients presenting with a transient amnestic syndrome.

In the absence of any similar study, we aimed to determine whether there has been any evolution of the clinical spectrum of TGA in routine clinical practice since the original report by comparing the clinical features of TGA ascertained in two independent cohort studies in Oxfordshire, UK [Hodges & Warlow 1977–1987 1, 2 versus Oxford Vascular Study (OXVASC) 2002–2018] and by validating the results in another independent contemporary TGA cohort in Italy [Northern Umbria TGA registry (NU)]. We also aimed to study the frequency and prognosis of short‐duration TGA.

## Methods

Consecutive patients with first‐in‐study‐period suspected TGA were prospectively collected from OXVASC and NU. Both studies received ethical approval (OXVASC, OREC A−05/Q1604/70; NU, CEAS‐12976/18) and informed consent was provided. We also extracted data from the original TGA cohort reported by Hodges and Warlow (Oxford cohort) 2.

The original Oxford cohort consisted of 114 patients diagnosed with TGA either as part of the Oxfordshire Community Stroke Project or at the Department of Neurology in Oxford between 1977 and 1987. Patients were followed up for a mean duration of 34.8 months 2.

The current Oxford cohort (OXVASC) is an ongoing population‐based study of all incident and recurrent vascular events in a population of 92 728 individuals registered with 100 general practitioners in nine general practices in Oxfordshire, UK, which overlaps the general practice population of the Oxfordshire Community Stroke Project. The study methods have been reported previously 10. As part of the study methods, all participating general practitioners were asked to refer any patient with suspected transient neurological attacks to the daily rapid‐access transient ischaemic attack/stroke clinic. Daily searches of the local emergency department (ED) register and admissions to medical, stroke or neurology wards were also performed to ascertain cases that were admitted. All cases were reviewed by the senior study neurologist (P.M.R.) and only patients with a final diagnosis of TGA from 1 April 2002 to 31 October 2018 were included in the current study.

The NU is a TGA registry including all patients diagnosed with TGA seen in the S. Maria della Misericordia Hospital, Perugia between 2002 and 2018. In Italy, the public National Health System allows direct referral to the ED for any suspected TGA and, as a result, almost all suspected TGA cases were managed at ED or admitted as inpatients. All cases with suspected TGA were assessed by a neurologist as soon as possible and only patients with a definite diagnosis of TGA were included in the current study.

In both OXVASC and NU, patients with suspected TGA were assessed as soon as possible and the standard clinical diagnostic criteria for TGA were used 2. Further investigations were left to the treating physician but patients routinely had brain imaging [computed tomography or magnetic resonance imaging (MRI)] and EEG was performed in cases where TEA was suspected, with TEA diagnosed according to standardized criteria, including (i) a history of recurrent witnessed episodes of transient amnesia, (ii) normal cognitive function during the episode apart from memory impairment and (iii) evidence for a diagnosis of epilepsy including concurrent onset of other clinical features of epilepsy, clear‐cut response to anticonvulsant treatment or epileptiform abnormalities on EEG 6.

In order to study the full spectrum of the disease and to represent routine clinical practice, all patients with confirmed TGA were included in the analysis and we did not limit the analyses only to those who had MRI and EEG performed. Demographic data, previous medical history including history of TGA and clinical features of the presenting episode (e.g. trigger, duration of anterograde amnesia, duration of retrograde amnesia, accompanying symptoms) were collected from face‐to‐face interview, supplemented by review of medical records.

All patients were followed up by a combination of face‐to‐face interview, telephone interview and administrative follow‐up to identify any major cardiovascular events (MaCEs), recurrent TGA, seizure/epilepsy or death. MaCEs included a composite outcome of non‐fatal stroke, non‐fatal acute coronary syndrome or death from cardiovascular causes. In OXVASC, follow‐ups were usually performed at 1 month, 3 months, 6 months, 1 year, 5 years and 10 years, and all patients presenting with stroke or acute coronary syndrome would also be prospectively identified by the ongoing daily ascertainment 10. Stroke was defined as a new symptomatic neurological deterioration lasting at least 24 h that was not attributable to a non‐stroke cause. Acute coronary syndrome included myocardial infarction with or without ST‐segment elevation or unstable angina followed by urgent catheterization.

### Statistical analysis

Continuous variables are presented as means and SD and categorical variables as *n* (%). Kaplan–Meier survival analyses were first used to present the time‐course and risks of vascular events during follow‐up, censored at death or 31 October 2018. We then calculated the annual rates of MaCE, recurrent TGA and seizure/epilepsy using Poisson distribution.

We also compared the baseline characteristics and clinical features of TGA ascertained in the two independent cohort studies in Oxfordshire, UK (Oxford cohort 1, 2 vs. OXVASC). We then compared the clinical feathers of TGA in two contemporary TGA cohorts (OXVASC, UK vs. NU, Italy). To characterize the clinical features and prognosis of short‐duration TGA, we compared the demographic data, risk factors, clinical presentation and long‐term risks of MaCE, recurrent TGA or seizure/epilepsy in patients with TGA lasting for < 1 h versus those lasting for ≥ 1 h in OXVASC and NU, respectively.

All statistical analyses were carried out using IBM SPSS Statistics for Windows, Version 25.0. Armonk, NY, USA: IBM Corp.

## Results

Overall, 639 patients with TGA were included (114 Oxford cohort 2 100 OXVASC, 425 NU). During the 16 years of OXVASC and NU, nine patients were diagnosed with TEA and were thus excluded from the analyses. Overall, in OXVASC and NU combined, 345 patients (65.7%), none of whom displayed epileptic features, underwent EEG.

Compared with the original Oxford cohort, patients in OXVASC were older [mean ± SD age (years): 68.2 ± 8.9 vs. 62.3 ± 8.5 in the Oxford cohort, *P* = 0.001; Table 1], more likely to have diagnosed hypertension [*n* (%): 48 (48.0%) vs. 25 (21.9%), *P* < 0.001; Table 1] and less likely to be current smokers [*n* (%): 5 (5.0%) vs. 17 (14.9%), *P* = 0.02; Table 1]. The baseline characteristics of OXVASC and NU were broadly similar, although patients in NU were younger and had a slightly higher prevalence of hypertension (Table1). However, the frequency of treated hypertension did not differ between OXVASC and NU [*n* (%): 44 (44.0%) vs. 196 (46.1%) for NU, *P* = 0.70; Table 1].

**Table 1:** Baseline characteristics in the 1977–1987 Oxford cohort 2 versus Oxford Vascular Study (OXVASC) versus Northern Umbria transient global amnesia (TGA) registry (NU)

	Oxford cohort (*n* = 114)	OXVASC cohort (*n* = 100)	_OXVASC vs. Oxford _ *_P_*_‐value_	NU cohort (*n* = 425)	_OXVASC vs. NU_ *_P_*_‐value_
Demographics					
Study period	1977–1987	2002–2018		2002–2018	
Age (years)	62.3 ± 8.5	68.2 ± 8.9	0.001	64.4 ± 9.5	0.01
Male sex	69 (60.5%)	49 (49.0%)	0.15	174 (40.9%)	0.14
Clinical characteristics of TGA					
Retrograde amnesia^a^	40 (35.1%)	47 (47.0%)	0.07	201 (47.3%)	0.96
Duration of retrograde amnesia (h)	1 (0.75–10)	48 (1–8600)	0.04	48 (1–672)	0.10
Abnormal neurological signs^b^	14 (12.3%)	5 (5.0%)	0.14	11 (2.6%)	0.21
Autonomic features (nausea, vomiting, sweating)	11 (9.6%)	6 (6.0%)	0.33	44 (10.4%)	0.18
Cardiovascular risk factors					
Hypertension	25 (21.9%)	48 (48.0%)	<0.001	257 (60.5%)	0.02
Treated hypertension	20 (17.5%)	44 (44.0%)	<0.001	196 (46.1%)	0.70
Dyslipidaemia	–	34 (34.0%)	–	178 (41.9%)	0.15
Diabetes	2 (1.7%)	2 (2.0%)	0.90	25 (5.9%)	0.11
Smoking					
Ever	56 (49.1%)	41 (41.0%)	0.23	150 (35.3%)	0.29
Current smoker	17 (14.9%)	5 (5.0%)	0.02	49 (11.6%)	0.05

a Retrograde amnesia represents clear loss of memory before the acute onset of TGA episode (with its duration expressed in h).

b Included incoordination (mild in all cases), unilateral facial weakness, unilateral motor deficit, postural tremor, nystagmus, speech disturbances, reflex asymmetry and extensor plantar response. All abnormalities detected during neurological examination were either known to antedate TGA or were due to unrelated or non‐neurological causes. Data are given as mean ± SD, *n* (%) and median (range).

**Figure 1:**
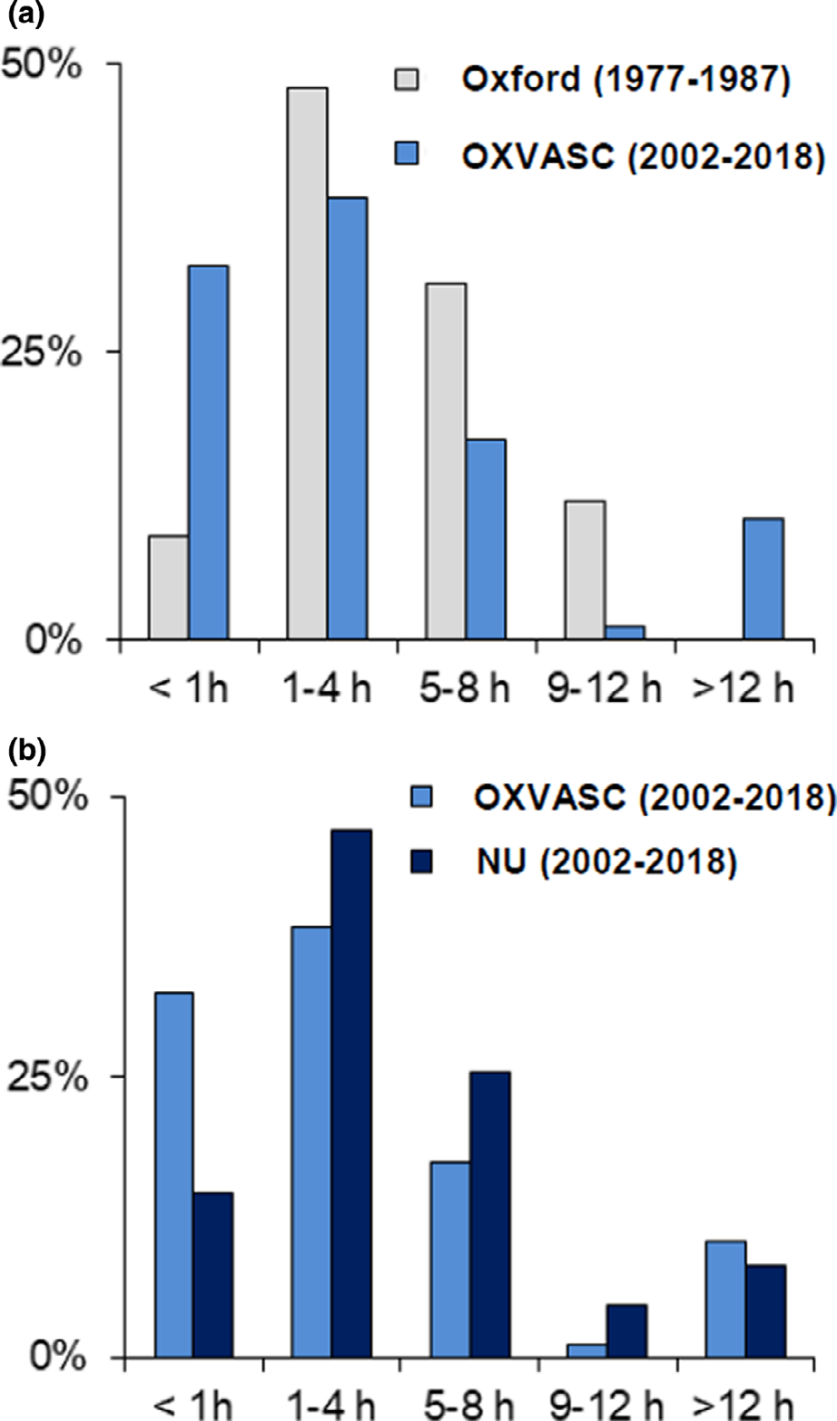
Frequency of anterograde memory deficit of different duration in (a) Oxford cohort versus Oxford Vascular Study (OXVASC) versus (b) Northern Umbria transient global amnesia registry (NU).

Clinical characteristics of the TGA in the three cohorts are listed in Table 1. As expected, abnormal neurological signs or autonomic features were similarly uncommon in all three cohorts (Table 1). However, retrograde amnesia was documented marginally more frequently in OXVASC than in the Oxford cohort (47.0% vs. 35.1% for the original Oxford cohort, *P* = 0.07; Table 1), but was similar between OXVASC and NU (47.0% vs. 47.3% for NU; Table 1). There were significantly more cases with TGA anterograde amnesia lasting < 1 h in OXVASC than in the original Oxford cohort (32.0% vs. 8.8%, *P* < 0.001; Fig. 1). Although TGA cases lasting < 1 h were less commonly seen in NU than in OXVASC (11.8% vs. 32.0% for OXVASC, *P* < 0.001; Fig. 1), they were still marginally more frequent than in the original Oxford cohort (11.8% vs. 8.7%, *P* = 0.23; Fig. 1). It is of note that there was some evidence of digit preference with 34 (8.0%) of the patients in NU reporting TGA episodes lasting for exactly 1 h versus 5 (5.0%) in OXVASC.

Table 2 compares the baseline risk factors and clinical features of patients with TGA lasting < 1 h versus ≥ 1 h in OXVASC and NU, respectively. In OXVASC, patients with TGA lasting < 1 h did not differ from those with TGA lasting ≥ 1 h in age, sex, vascular risk factors, clinical features or neurological examination at onset (Table 2). The same was found in the NU cohort, although patients with TGA lasting < 1 h reported a higher prevalence of dizziness than those with TGA lasting ≥ 1 h in the NU cohort (Table 2).

**Table 2:** Differences in clinical features and risk factors between short‐lasting (<1 h) and typical (≥1 h) transient global amnesia (TGA) stratified by cohorts

	NU cohort (*n* = 425)		*P*_1_	OXVASC cohort (n = 100)		*P*_2_
	Typical (≥1 h) (*n* = 375)	Short‐lasting (<1 h) (*n* = 50)		Typical (≥1 h) (*n* = 68)	Short‐lasting (<1 h) (*n* = 32)	
Age at event (years)	64.5 ± 9.2	63.3 ± 11.5	0.39	67.6 ± 9.2	69.4 ± 8.4	0.32
Male sex	155 (41.3%)	19 (38.0%)	0.65	36 (52.9%)	13 (40.6%)	0.25
Clinical features						
Wake‐up onset	41 (10.9%)	5 (10%)	0.99	13 (19.1%)	1 (3.1%)	0.03
Patchy recollection during the event	38 (10.5%)	7 (14.6%)	0.46	11 (16.2%)	7 (21.9%)	0.49
Other symptoms	61 (16.3%)	17 (34%)	0.01	15 (22.1%)	10 (31.3%)	0.32
Dizziness	2 (0.5%)	4 (8%)	0.002	4 (5.9%)	2 (6.3%)	0.94
Findings on neurological examination	8 (2.1%)	3 (6%)	0.13	4 (5.9%)	1 (3.1%)	0.58
Retrograde amnesia	179 (47.7%)	22 (44%)	0.37	31 (45.6%)	16 (50.0%)	0.68
Cardiovascular risk factors						
Hypertension	227 (60.5%)	30 (60%)	0.99	30 (44.1%)	18 (56.2%)	0.26
BP >180/100 mmHg at TGA^a^	32 (10.2%)	7 (15.2%)	0.31	4 (5.9%)	1 (3.1%)	0.56
SBP (mmHg)^a^	152.3 ± 24.8	148.7 ± 29.2	0.36	148.0 ± 24.0	146.4 ± 20.7	0.76
DBP (mmHg)^a^	88.1 ± 12.0	85.5 ± 13.7	0.18	85.3 ± 11.5	84.2 ± 15.9	0.74
Dyslipidaemia	152 (40.5%)	26 (52%)	0.13	24 (35.3%)	10 (31.2%)	0.69
Total cholesterol level (mg/dL)^b^	212.4 ± 37.1	209.6 ± 35.7	0.70	218.3 ± 35.4	224.1 ± 40.7	0.58
LDL‐cholesterol level (mg/dL)^b^	130.9 ± 35.8	126.5 ± 39.4	0.72	132.0 ± 26.6	139.5 ± 25.2	0.64
Diabetes	19 (5.1%)	6 (12%)	0.10	1 (1.5%)	1 (3.1%)	0.58
Smoking (current)	44 (11.7%)	5 (10.2%)	0.91	26 (38.2%)	15 (46.9%)	0.41
Ex‐smoker	90 (24%)	11 (22.4%)		24 (35.3%)	12 (37.5%)	

BP, blood pressure; DBP, diastolic BP; LDL, low‐density lipoprotein; SBP, systolic BP. Data are given as mean ± SD and *n* (%).

a At first assessment, data available for 85.4% of the whole cohort [84.4% Northern Umbria TGA registry (NU) cohort, 88.7% Oxford Vascular Study (OXVASC) cohort].

b At first assessment, data available for 57.8% of the whole cohort (56.6% NU cohort, 66.3% OXVASC cohort).

During follow‐up of 3905 patient‐years, there were 46 MaCEs, 39 recurrent TGA and six seizure/epilepsy in OXVASC and NU (Table 3). In OXVASC, annual risk of any MaCE was 0.6% [95% confidence interval (CI), 0.1–3.2] after a TGA episode lasting < 1 h versus 1.6% (95% CI, 0.6–3.3) after a TGA episode lasting ≥ 1 h (*P* = 0.35). Similarly, in NU, risks of any MaCE did not differ between those with episodes lasting < 1 h versus ≥1 h (1.8% vs. 1.2% for ≥ 1 h, *P* = 0.42). Moreover, no differences in risks of recurrent TGA or seizure/epilepsy were found between TGA lasting < 1 h versus TGA lasting ≥ 1 h in OXVASC or NU (Table 3). It is notable that risks of seizure/epilepsy were also much lower in OXVASC and NU than in the original Oxford cohort (Oxford cohort 2.5%, 95% CI, 1.1–4.7 vs. OXVASC 0.3%, 95% CI, 0.1–1.0 vs. NU 0.1%, 95% CI, 0.0–0.3; OXVASC vs. Oxford, *P* = 0.004; OXVASC vs. NU, *P* = 0.31).

**Table 3:** Differences in outcomes between short‐lasting (<1 h) and typical (≥1 h) transient global amnesia (TGA) stratified in the two contemporaneous cohorts

	NU cohort				*P*_1_	OXVASC cohort				*P*_2_
	Typical (≥1 h) (*n* = 375)		Short‐lasting (<1 h) (*n* = 50)			Typical (≥1 h) (*n* = 68)		Short‐lasting (<1 h) (*n* = 32)		
	*n*	Annual rate (95% CI)	*n*	Annual rate (95% CI)		*n*	Annual rate (95% CI)	*n*	Annual rate (95% CI)	
MaCE	33	1.2 (0.8–1.7)	5	1.8 (0.6–4.0)	0.42	7	1.6 (0.6–3.3)	1	0.6 (0.1–3.2)	0.35
Seizure/epilepsy	4	0.1 (0.0–0.3)	0	0 (/)	0.64	1	0.2 (0.0–1.2)	1	0.6 (0.0–3.1)	0.55
TGA recurrence	26	0.9 (0.6–1.3)	4	1.2 (0.3–3.0)	0.57	7	1.6 (0.6–3.3)	2	1.1 (0.1–3.8)	0.64

CI, confidence interval; MaCE, major cardiovascular event, including death from cardiovascular cause, non‐fatal myocardial infarction or non‐fatal stroke; NU, Northern Umbria TGA registry; OXVASC, Oxford Vascular Study.

## Discussion

Using data from three independent cohorts of patients with TGA, we showed for the first time that short‐duration TGA episodes (i.e. <1 h) are not uncommon and have become more recognized over the last four decades. Reassuringly, the clinical features and long‐term prognosis of short‐duration TGA did not differ from more typical episodes lasting ≥ 1 h.

Our findings suggest that there has been an expansion in clinical phenotypes of TGA since the introduction of the diagnostic criteria in 1990, particularly in Oxfordshire, UK 2. This is perhaps not surprising and has also been reported in other neurological diseases 11, 12. The apparently rising prevalence of ‘milder’ or ‘atypical’ cases is most likely to be related to increasing awareness and recognition of the disease by physicians. The design of this study allowed comparison of the temporal trends of clinical features in two cohort studies from the same underlying population (Oxfordshire) and then confirmation of the findings in a third independent study, accounting for regional variations. Although the prevalence of TGA lasting < 1 h appeared to be lower in NU versus OXVASC, possibly due to some digit preference in reporting in NU, it was still higher in NU than in the Oxford cohort 2, further supporting an expansion in TGA phenotype. Moreover, the benign long‐term prognosis of TGA episodes in both NU and OXVASC cohorts corroborates that short‐lasting TGA is becoming increasingly common but is still benign.

Transient amnesic episodes lasting for < 1 h are challenging to diagnose and are often considered as a typical feature of TEA. However, we found that short‐duration TGA (i.e. <1 h) was much more common than TEA. In OXVASC and NU combined, 82 TGA cases were found to be < 1 h versus three of the nine TEA cases. Moreover, we showed that TGA cases lasing < 1 h had similar risk factor profiles, clinical features and long‐term prognosis compared with more typical TGA cases lasting ≥ 1 h. Therefore, short‐duration TGAs should also be managed as typical TGAs and clinicians can avoid over‐requesting of additional diagnostic tests.

We also showed that the absolute risks of seizure after TGA in the two contemporary cohorts were very low and were much lower than in the original Oxford cohort. This finding has implications on management of these patients. In the UK, the Driver and Vehicle Licensing Agency requires appropriate investigations to exclude seizure after a brief episode of amnesia (https://www.gov.uk/transport). However, our results highlight that, given the low risk of seizure even in those with TGA episodes lasting < 1 h, routine MRI or EEG may not be justified even in those with episodes of short duration. Moreover, these more up‐to‐date risk estimates should also be taken into account for patient consultation and for future regulations/policies for driving after TGA.

The strengths of our study include that we were able to compare the temporal trends in studies from the same population (i.e. Oxfordshire) and to compare the results in a third larger independent cohort in Italy, accounting for any confounding by national differences. However, the study also has limitations. Firstly, although attempts were made to make both cohorts inclusive, cases could still be missed. In OXVASC, those patients with typical TGA (i.e. lasting ≥ 1 h) might not have been referred to the OXVASC study clinic as general practitioners might be more comfortable making the diagnosis themselves. Hence, we may have overestimated the prevalence of TGA lasting < 1 h in OXVASC. On the contrary, in Italy, patients were more likely to present to the ED directly. Patients with very brief episodes might decide not to go the hospital in the first place, particularly if they were alone when the episode occurred. Therefore, the true prevalence of TGA lasting < 1 h might be underestimated in the NU cohort. Second, we did not routinely perform brain MRI in all patients with TGA and hence we were not able to compare the prevalence of imaging findings in TGA cases lasting < 1 h versus those lasting ≥ 1 h. However, it was reassuring that the risks of MaCEs were low in both cohorts and therefore misdiagnosis of ischaemic events for TGA is unlikely. Third, we did not routinely perform EEG. However, 66% of patients in OXVASC and NU combined had EEG, 59% of those with TGA lasting < 1 h. No EEG revealed epileptiform abnormalities and no differences were found between patients undergoing and those not undergoing EEG. Although less than 5% of the patients underwent EEG during the TGA episode, the lack of epileptiform abnormalities in these patients at onset, together with the systematic ascertainment of any recurrent TGA‐like symptoms and the very low risk of seizure/epilepsy during follow‐up, suggests that substantial misdiagnosis of TEA for TGA is also unlikely. Fourth, we were only able to compare the clinical characteristics and outcomes of TGA lasting < 1 h versus TGA lasting ≥ 1 h in the OXVASC and NU cohort as the equivalent data from the original Oxford cohort were not available. Fifth, some patients may not have the whole TGA episode witnessed and hence there could be underestimation of the true duration of some TGA episodes. However, the way in which duration was recorded in the current study reflects routine clinical practice, where clinicians base their judgement on the reported duration from the witnessed episode. Finally, the TGA diagnosis in both OXVASC and NU was made after careful neurological ascertainment. Therefore, our results may not be generalizable to settings where TGA is largely managed by non‐neurology specialties.

In conclusion, we showed that short‐duration TGA episodes (i.e. <1 h) are not uncommon and have become more recognized in the last four decades. Reassuringly, the clinical features and long‐term prognosis of short‐duration TGA did not differ from more typical episodes lasting ≥ 1 h and therefore they should still be managed in the same way as those otherwise typical TGAs.

## Disclosure of conflicts of interest

The authors declare no financial or other conflicts of interest.
